# The Effect of Cerebrolysin on the Predictive Value of Baseline Prognostic Risk Score in Moderate and Severe Traumatic Brain Injury

**DOI:** 10.25122/jml-2020-0146

**Published:** 2020

**Authors:** Codruta Birle, Dana Slavoaca, Ioana Muresanu, Diana Chira, Vitalie Vacaras, Adina Dora Stan, Constantin Dina, Stefan Strilciuc

**Affiliations:** 1. Department of Neurosciences, “Iuliu Hatieganu” University of Medicine and Pharmacy, Cluj-Napoca, Romania; 2. “RoNeuro” Institute for Neurological Research and Diagnostic, Cluj-Napoca, Romania; 3. Neurology Clinic, Cluj Emergency County Hospital, Cluj-Napoca, Romania; 4. Department of Radiology, “Ovidius” University, Faculty of Medicine, Constanta, Romania

**Keywords:** Traumatic brain injury, prognostic, cognitive outcome, Cerebrolysin, AIS - Abbreviated Injury Scale, BPRS - Baseline Prognostic Risk Score, CTT - Colour Trails Test, GCS - Glasgow Coma Scale, GOSE - Glasgow Outcome Scale Extended, HADS - Hospital Anxiety and Depression Scale, IMPACT - The International Mission on Prognosis and Analysis of Clinical Trials in TBI, MMSE - Mini Mental State Examination, MRC CRASH - Medical Research Council Corticosteroid Randomization after Significant Head Injury, PSI DSC - Processing Speed Index Digit Symbol Coding, PSI SS - Processing Speed Index Symbol Search, PTA - post-traumatic amnesia, TBI - Traumatic Brain Injury, WAIS III - Wechsler Adult Intelligence Scale Third Edition.

## Abstract

Cognitive dysfunction is a significant complaint among patients after moderate to severe traumatic brain injury (TBI), with devastating consequences on functional recovery and quality of life. Prognostic models allow a better assessment and management of neurotrauma patients.

The aim of the study was to demonstrate the predictive value of the Baseline Prognostic Risk Score (BPRS) in moderate to severe TBI, in a sample of patients treated with neurotrophic factors.

Eighty patients with moderate-severe TBI from the CAPTAIN II study were included in secondary data analysis. Patients received active treatment with Cerebrolysin, 50 mL per day for ten days, followed by two treatment cycles with 10 mL per day for ten days. BPRS was determined on admission; the age was recorded, and patients were evaluated using the following neurocognitive tests: Mini-Mental State Essay (MMSE), Wechsler Adult Intelligence Scale-Third Edition Processing Speed Index (WAIS-III PSI) and Stroop Colour Word Test-Victoria Version at 10, 30 and 90 days. Hierarchical regression analysis was performed to investigate the unique predictive value of BPRS on cognitive evolution, independent of age. BPRS independently predicted scores on the WAIS-III PSI DSCales and the Word subscale of the Stroop Colour Word Test at 90 days. Age was a significant predictor for all the investigated scales at 10, 30, and 90 days.

This study demonstrates the predictive value of a validated prognostic model (BPRS) for medium-term neurocognitive outcomes in a sample of moderate-severe traumatic brain injury treated with neurotrophic factors.

## Introduction

Traumatic brain injury remains a significant health issue as 27.08 million new cases are reported each year, according to the Global Burden of Disease Study 2016 [1]. In the European Union, 1.5 million hospital admissions per year, and 57.000 deaths are caused by traumatic brain injury (TBI) [2]. Despite recent advances in managing trauma patients, TBI remains a leading cause of long-lasting disability and functional impairment. The primary focus for the management of these patients and their outcome remains mortality and physical disability at six months. However, cognitive sequelae pose a major burden on victims, their families, and society [3]. Cognitive dysfunction is a strong predictor of disability following TBI and is a primary complaint among moderate to severe TBI survivors.

Numerous factors have been associated with cognitive outcomes post-TBI [4, 5]. The severity of the injury is defined by the initial Glasgow Coma Score (GCS), duration of post-traumatic amnesia (PTA) and computed tomography (CT) scan lesions [6], and is a crucial predictor of the outcome. Premorbid demographic factors include age, gender, education level, occupation, intelligence coefficient, and genotype [7]. The extent and rate of recovery vary among patients with TBI. Some studies show that cognitive disturbances resolve entirely in three to six months in approximately 80-85% of people after mild TBI, while others imply that one-third of the patients with mild TBI will have persistent functional impairment at three months [8]. In moderate to severe TBI, persistent cognitive deficits are present in 65% of the patients. Executive function, attention, and memory are cognitive domains that are affected most in TBI. In 2008, Christensen et al. stated that for some cognitive functions like memory, executive function and manual dexterity, the degree of recovery is higher in the first 2 to 5 months than in the 5 to 12 months post-injury [9].

Age is a significant predictor of cognitive function, younger age being associated with more favorable neuropsychological outcomes [10]. Cross-sectional and longitudinal studies demonstrated more unsatisfactory cognitive performance in trauma patients over 50 years old [11]. These findings may be associated with reduced cognitive reserve, reduced neuroplasticity, or increased synaptic pruning in the elderly. Reduced volume of the superior temporal and parietal regions was observed in older TBI patients when compared to healthy controls[12]. Long-term cognitive recovery may be reduced in older patients due to a synergetic effect between age-related cognitive decline and injury-related pathophysiological mechanisms [13].

Several prognostic models have been developed to address the multi-faceted evolution of TBI patients, including cognitive outcomes. The International Mission on Prognosis and Analysis of Clinical Trials in TBI (IMPACT) [11] and Medical Research Council Corticosteroid Randomization after Significant Head Injury (MRC CRASH) [14] are two scales widely used in practice and clinical studies for their well-established predictive value. The Baseline Prognostic Risk Score (BPRS), developed by Hukklehoven et al., estimates the risk of mortality and unfavorable outcome at six months [15]. In the CAPTAIN I [16] and CAPTAIN II [17] studies, this scale was used to assess the heterogeneity of the study population.

Several pharmacological neuroprotective agents have been studied in trauma patients, some of them showing a beneficial effect regarding the functional outcome [18]. Confirming previous data [19]–[21], the CAPTAIN II study demonstrated that Cerebrolysin, a multimodal neurotrophic agent, improved global outcome, cognitive speed, attention, and depression at 90 days post-TBI as measured by an ensemble of scales addressing each functional domain.

The purpose of our study was to analyze the role of BPRS in predicting short- and medium-term cognitive outcome in moderate to severe TBI patients receiving active treatment with neurotrophic factors. The influence of age on cognitive recovery, under the same conditions, was also investigated.

## Material and Methods

Data were extracted from the CAPTAIN II Study database [17]. CAPTAIN II is a single-center, prospective, randomized, double-blind, placebo-controlled clinical trial. The protocol was approved by the Ethics Committee of the University of Medicine and Pharmacy in Cluj-Napoca, Romania (No. 714/07.03.2013), and it is available in the ISRCTN registry (No. 17097163). Patients with moderate to severe traumatic brain injury were randomized either to the treatment group receiving pharmacological treatment with Cerebrolysin or to the placebo group receiving saline solution. The trial lasted for 90 days after the injury.

The initial evaluation of the neurological and general status consisted of three scales: Glasgow Coma Scale (GCS) [22], Abbreviated Injury Scale (AIS) [23], and Baseline Prognostic Risk Factors (BPRS) [15]. BPRS is a prognostic model, designed to stratify the population of the study. The items included are age, motor score, Marshall computed tomography classification, pupillary reactivity, hypoxia, hypotension, and traumatic subarachnoid hemorrhage.

Patients had a multidimensional assessment based on outcome measures for different domains:

a. Global level of function: Glasgow Outcome Scale Extended (GOSE) [24] and Early Rehabilitation Barthel Index (ERBI);
b. Neurocognitive impairment: Mini-Mental State Essay (MMSE) [25], Colour Trails Test (CTT) [26], Wechsler Adult Intelligence Scale-Third Edition Processing Speed Index (WAIS-III PSI) [27], Digit Span Forward and Digit Span Backwards[27], Stroop Colour Word Test -Victoria Version [28];
c. Psychological status - Hospital Anxiety and Depression Scale (HADS) [29]. Ensembles of the evaluation scales were performed at 10, 30, and 90 days. MMSE, PSI, Stroop Colour Word Test constituted the core of the neurocognitive evaluation for each visit.

Data from the patients in the Cerebrolysin group were analyzed to establish the predictive value of BPRS under active pharmacological treatment. 

## Statistical methods

In order to investigate the unique predictive value of BPRS on cognitive evolution independent of age, hierarchical regression analysis was performed using neuropsychological scales, MMSE, PSI, Stroop Colour Word Test as dependent variables. Two different models were used to determine how much the independent variables, BPRS and age, predict the dependent variables. For Step 1, r2 is the proportion of explained variance of dependent variables by the model (age). For Step 2, r2 represents the proportion of explained variance of dependent variables by the model (BPRS), when the beta regression coefficient (β) is calculated for age. Regressions were generated through SPSS software. 

## Results

Of the 142 patients enrolled in the CAPTAIN II study, 80 were in the active treatment group. 90% were male patients (N=70), with a mean age of 46.4 years (SD=17.1). The mean BPRS was 2.6 (SD=1.8).

The results of the neurocognitive scores are presented in Table 1.

**Table 1:** Assessment of Global Cognitive Function, Executive Function and Attention.

	10 days	30 days	90 days
	Mean (SD)	Mean (SD)	Mean (SD)
**MMSE**	26.6 (2.8)	28.3 (1.6)	29.1 (1.4)
**PSI SS**	22.1 (6.5)	28.9 (6.5)	30.6 (6.9)
**PSI DSC**	45.9 (11.3)	52.0 (11.2)	53.3 (12.2)
**Stroop Dots**	21.7 (5.0)	17.6 (4.8)	16.1 (5.8)
**Stroop Word**	29.8 (5.7)	24.6 (5.6)	22.0 (8.1)
**Stroop Colour**	53.8 (14.8)	48.7 (14.8)	45.5 (14.4)

Note: MMSE: Mini-Mental State Examination; PSI DSC: Processing Speed Index -Digit Symbol Coding; PSI SS: Processing Speed -Symbol Search; Stroop Dots -Stroop Colour Word Test VTS Dots Subtest, Stroop Word: Stroop Colour Word Test VTS Words Subtest; Stroop Colours: Stroop Colour Word Test VTS Colours Subtest.

Age was a significant predictor of MMSE at 10, 30 and 90 days, whereas BPRS was a significant predictor of MMSE scores at 90 days (p = 0.001; r2 = .14) but not at 10 or 30 days. BPRS alone explains 14% of the variance of the MMSE scores at 90 days. As shown in Table 2, lower BPRS scores predicted higher MMSE scores.

**Table 2:** Prognostic value of BPRS and Age for MMSE.

	MMSE 10d	MMSE 30d	MMSE 90d
**Step 1: Age**	r^2^ = 0.13	r^2^ = 0.13	r^2^ = 0.10
	p = 0.002	p = 0.002	p = 0.007
**Step 2: BPRS**	non-sig	non-sig	r^2^ = 0.14
			β = -0.59
			p = 0.001

Age was a significant predictor of PSI DSC at 10, 30, and 90 days, as shown in Table 3. BPRS was a significant predictor of PSI DC scores at 90 days (p = 0.026; r2 = .04) but not at 10 or 30 days. BPRS alone explains 4% of the variance of the PSI DC scores at 90 days.

**Table 3:** Prognostic value of BPRS and Age for PSI DSC.

	PSI DSC 10d	PSI DSC 30d	PSI DSC 90d
**Step 1: Age**	r^2^ = 0.32	r^2^ = 0.30	r^2^ = 0.33
	p = 0.000	p = 0.000	p = 0.000
**Step 2: BPRS**	non-sig	non-sig	r^2^ = 0.04
			β = -0.33
			p = 0.026

BPRS was also a significant predictor of the PSI SS scores at 90 days, explaining 6% of the variance (r2= 0.06, β=- 0.40, p=0.010). Age predicted PSI SS scores at 10 (r2= 0.33, p=0.000), 30 (r2= 0.17, p=0.000) and 90 days (r2= 0.26, p=0.000) and explained 17 to 33% of the variance. 

For subscales Dots and Colours of the Stroop Colour Word Test, BPRS did not have a predictive value. However, age was a significant predictor of scores for both subscales at all visits (Table 4).

**Table 4:** Prognostic value of BPRS and Age for Stroop Colour Word Subtests.

		10d	30d	90d
**Stroop Dots**	**Step 1: Age**	r^2^ = 0.32	r^2^= 0.33	r^2^ = 0.34
		p = 0.000	p = 0.000	p = 0.000
	**Step 2: BPRS**	non-sig	non-sig	non-sig
**Stroop Words**	**Step 1: Age**	r^2^ = 0.19	r^2^ = 0.23	r^2^ = 0.20
		p = 0.000	p = 0.000	p = 0.000
	**Step 2: BPRS**	non-sig	non-sig	r^2^ = 0.04
				β = 0.32
				p = 0.046
**Stroop Colours**	**Step 1: Age**	r^2^ = 0.30	r^2^ = 0.20	r^2^ = 0.15
		p = 0.000	p = 0.000	p = 0.000
	**Step 2: BPRS**	non-sig	non-sig	non-sig

BPRS was a significant predictor of Stroop Word subtest scores at 90 days (Table 4). The higher scores of BPRS were predictors for higher scores on this scale (β= 0.32). Age is a significantly predicted score at 10, 30, and 90 days.

## Discussion

BPRS was initially validated on selected populations from two studies on Tirilazad in moderate and severe TBI patients: International and North American multi-center (phase III) randomized control studies [15]. The two outcome measures were mortality and unfavorable outcome at 6 months post-injury, with GOS as a measure for an unfavorable outcome. Since there was no statistically significant difference between the Tirilazad and the placebo-treated group for the primary outcome measure, BPRS was practically assessed in TBI patients without active intervention, using a global outcome scale measure. The purpose of this study was to investigate if BRPS could predict cognitive recovery in patients with active neurotrophic treatment, independently of age.

In the CAPTAIN II study [17], an ensemble of scales measuring global function outcome and the neurocognitive outcome was used to prove the statistically significant effect of Cerebrolysin in the first primary endpoint (Day 90). Therefore, the predictive value of age and BPRS significantly influenced by the treatment in our current study.

The results of the current study demonstrated the good predictive value of BPRS at 90 days for several cognitive outcomes measures WAIS PSI DSC, WAIS PSI SS and Stroop Colour Word Test- Word subscale. 

Major contributors to BPRS are age, GCS motor score, pupilar reactivity, and CT lesions assessed according to the Marshall classification [30].

Our results show that in TBI patients receiving Cerebrolysin treatment, age is an important predictor of global cognitive function measured by MMSE, of the speed of processing measured by PSI DSC and PSI SS, and of attention measured by Stroop Color Word subtests, at 10-, 30- and 90-days assessments. Underactive treatment, age retains the same impact on processing speed, executive function, and memory, as previously demonstrated in longitudinal studies investigating short-term cognitive recovery following TBI. Green et al. showed that younger age is associated with recovery from 2 to 12 months postinjury for both simple and complex speed of processing, but there is no effect on memory [31]. Older age was negatively associated with recovery for processing speed, but there was no evidence of an effect on memory or attention, suggesting a more selective therapy targeting the impaired domains. 

Marmarou et al. confirmed a strong relationship between GOS components and pupil reactivity and the 6-month outcome in TBI patients [32]. As the association of pre-hospital or first hospital motor scores and pupil reactivity to the outcome was not as strong due to the dynamic changes that occur from the moment of injury to study hospital admission, the authors recommended that study hospital enrolment values should be used for clinical trial design and prognosis. In a more recent study, it was shown that differences between the field and admission assessments did not influence the prognostic value significantly, suggesting that a valid prognosis could be made for a patient if any of the two assessments are available [33]. In our study, admission scores were used as the enrolment time-window was 4 hours from trauma. 

The predictive value of the Marshall CT classification was established in a large series of patients [34, 35]. Even though there are data implying that better discrimination can be obtained using the full individual CT characteristics, these are valid if the initial CT examination is performed within 4 hours after injury [35]. The worst CT scan obtained during the clinical course was shown to have a greater predictive value [36, 37]. However, for patient management and clinical studies, Marshall classification allows a good risk stratification.

Most prognostic models were validated using GOS/GOSE as an outcome measure. There was widespread concern that GOSE may be insufficiently sensitive and that it may have a limited ability to identify the mild disability and to capture the full spectrum of complaints that persist after TBI. In a 2017 study, Nelson et al. showed that in moderate to severe TBI patients, GOSE correlated with neurocognitive scores and especially with self-reported outcome measures, such as those designed to evaluate the emotional function and quality of life [38]. In our study, the lack of quality of life assessment limits the possibility to investigate the predictive value of BPRS in relation to the patients’ perspective.

## Conclusion

In this secondary data analysis, we described the prognostic value of BPRS for neurocognitive outcomes in patients after moderate and severe TBI, treated with Cerebrolysin. Given the high degree of heterogeneity between TBI survivors, this information may provide valuable insight for researchers looking to improve clinical trial designs by integrating instruments to control for baseline confounders.

## Conflict of Interest

The authors declare that there is no conflict of interest.
